# Care Recipient Diagnosis Moderates the Relationship Between Caregiver Worry and Vigilance

**DOI:** 10.1093/geroni/igab046.3466

**Published:** 2021-12-17

**Authors:** Katherine Craig, Shirit Kamil-Rosenberg, J Kaci Fairchild

**Affiliations:** 1 Veterans Affairs Palo Alto Health Care System, Palo Alto, California, United States; 2 VA Palo Alto Health Care System, Palo Alto, California, United States

## Abstract

Family members of persons diagnosed with dementia or a traumatic brain injury (TBI) are often relied upon to provide daily support to their care recipients. However, research on the differing experiences of caregivers based on care recipient diagnosis is limited. The aim of this study was to examine the impact of worry and feelings of vigilance among caregivers of people with cognitive impairment due to either TBI or dementia. This sample included 61 caregivers (88.5% female, mean age 57.3±15.5) of persons with either a TBI (n = 32) or dementia (n = 29). Worry was assessed with the Penn State Worry Questionnaire and Vigilance was assessed with the Caregiver Vigilance Scale. Linear regressions revealed that after controlling for age, care recipient diagnosis moderated the relationship between worry and caregiver vigilance. Specifically, worry was significantly associated with caregiver vigilance in those caring for someone with dementia; however, a similar relationship was not seen in those caring for someone with a TBI. This suggests caregivers of people with TBIs have a different experience of worry and vigilance than caregivers of people with dementia. These findings demonstrate the need for more research on the unique needs of caregivers of people with TBIs. Additionally, this research suggests interventions targeting worry may be particularly effective in supporting caregivers of people with TBIs.

